# Impact of Long-Term Tiotropium Bromide Therapy on Annual Lung Function Decline in Adult Patients with Cystic Fibrosis

**DOI:** 10.1371/journal.pone.0158193

**Published:** 2016-06-28

**Authors:** Claudia Brandt, Anja Thronicke, Jobst F. Roehmel, Alexander Krannich, Doris Staab, Carsten Schwarz

**Affiliations:** 1 Department of Pediatric Pneumology and Immunology, Cystic Fibrosis Center / Charité – Universitätsmedizin Berlin, Augustenburger Platz 1, 13353 Berlin, Germany; 2 Biostatistics Unit, Berlin Institute of Health, Charité – Universitätsmedizin Berlin, Augustenburger Platz 1, 13353 Berlin, Germany; University of Pittsburgh, UNITED STATES

## Abstract

**Background:**

Chronic lung disease is the leading cause of death in patients with Cystic Fibrosis (CF) and is often treated with bronchodilators. It is not known whether long-term tiotropium bromide treatment may have a positive impact on lung function.

**Methods:**

This retrospective cohort study estimated annual lung function decline utilizing longitudinal data for forced expiratory volume in 1 s (FEV1).

**Results:**

A total of 160 adult patients with CF were analyzed. The subjects treated for 24 months with tiotropium bromide had a significantly slower decline of mean annual change of FEV1 (treated: -0.3±4.0%; control: -2.3±5.0%; *p* = 0.0130). In patients with FEV1 ≥70% predicted, long-term tiotropium bromide treatment was associated with greater improvements in annual lung function decline (FEV1 ≥70% predicted: treated: +0.5±4.7%; control: -4.0±6.3%; *p* = 0.0132; FEV1 50–69% predicted: treated: -0.5±4.4%; control: -0.8±3.8%; *p* = 0.7142; FEV1 ≤49% predicted: treated: -0.6±3.4%; control: -2.4±4.8%; *p* = 0.0898).

**Conclusion:**

This study suggests that long-term tiotropium bromide treatment may be associated with reduced annual decline of FEV1 in patients with CF, particularly in adults with a mild degree of severity.

## Introduction

Cystic Fibrosis (CF) is a life-limiting autosomal recessive disorder that affects more than 70,000 individuals worldwide [1, 2]. Chronic airway infection, bronchiectasis, hypoxemia and hypercapnia as well as defective mucociliary clearance and bronchial and mucus obstruction are key features of the CF respiratory disease, resulting in a progressive lung function decline. Pulmonary insufficiency, with extensive airway destruction, is the main cause of death for patients with CF [3–7]. To describe disease severity and to monitor lung function in CF, the forced expiratory volume in 1 s (FEV1) is commonly used. FEV1 represents a marker of disease progression and a predictor of survival for patients with CF [5, 8–10]. To improve pulmonary function and lessen wheezing [4, 11] inhaled short- or long-acting bronchodilators have been routinely used in standard pulmonary CF therapy [11–14] but until now there is no approved indication for CF [12, 15]. The long-acting, inhaled anticholinergic bronchodilator tiotropium bromide (hereafter referred to as tiotropium) is approved for maintenance therapy in patients with chronic obstructive pulmonary disease (COPD) [16–18]. Several studies investigated the efficacy of tiotropium in patients with COPD. During ≥12 months of tiotropium treatment, improvements in lung function and measures of exacerbations were observed in patients with COPD [19–23].

With regard to CF, safety and efficacy of inhaled tiotropium were investigated in children and adults with CF in three randomized placebo-controlled multicenter trials with tiotropium treatment durations of ≤ 12 weeks [17, 24]. Dosages of 2.5 and 5 μg inhaled tiotropium showed consistent improvement in lung function [24, 25]. However, statistically significant differences in the measure of lung function were only reached in one study. Furthermore, the improvement in lung function was greater in patients who received the higher dose of 5 μg tiotropium [24]. Until now, long-term effects in CF as well as tiotropium dosages of 18 μg have not been studied. It is not known whether long-term tiotropium treatment may reduce lung function decline.

Here, longitudinal data of long-term 18 μg once daily inhaled tiotropium treatment in adult patients with different CF disease severities is presented. We hypothesized that long-term treatment with tiotropium for 24 months is effective in reducing lung function decline compared to non-tiotropium based CF standard therapy.

## Materials and Methods

### Study design

This was a retrospective cohort study of individuals with documented CF followed at the Cystic Fibrosis Center at the Charité–Universitätsmedizin Berlin (Berlin, Germany) from 2004 to 2014. Data were extracted from the patient registry database MUKO.doc (Axaris software and systeme GmbH, Ulm, Germany). Routinely collected longitudinal data on lung function and measures of exacerbation were available for 160 adults with confirmed diagnosis of CF [26]. Study protocol was approved by the local ethics committee of the Charité–Universitätsmedizin Berlin (EA2/117/14). All participants involved in this study provided written informed consents.

In this retrospective cohort study longitudinal data were used to evaluate modulatory effects of inhaled bronchodilator tiotropium (18 μg once daily) on pulmonary lung function and measures of exacerbation for 24 months. The primary endpoint was the mean annual decline of FEV1 during long-term tiotropium therapy compared to non-tiotropium treated control patients. The secondary endpoint included mean annual change of exacerbations. Pulmonary exacerbations were defined using criteria provided by Bilton et al. [27]. Patients with pulmonary exacerbations showed a recent change in at least two of the following parameters: change in sputum volume or color, increased cough, increased malaise, fatigue or lethargy, anorexia or weight loss, decrease in pulmonary function by 10% or more / radiographic changes and increased dyspnea [27]. In order to minimize bias and confounding, a matching procedure on potential confounders was performed. Appropriate controls for interpatient comparison were identified with regard to: pre-bronchodilator baseline lung function (FEV1_0M_ ± 5%; FEV1_0M_ defined as baseline FEV1 at the beginning (month 0) of 24 months observation period and before tiotropium treated patients started their tiotropium add-on therapy), age (± 5 years), gender, BMI, baseline concomitant medication and *Pseudomonas aeruginosa* colonization (Table 1, for demographic and baseline characteristics of the subgroups please see S1–S3 Tables). Baseline FEV1 (FEV1_0M_ / month 0) was defined as last measured FEV1 immediately before subjects started their tiotropium inhalation therapy. Follow-up FEV1 for 12 and 24 months were defined as mean of FEV1 between baseline/0 month to 12 months of observation period (month 12) as well as 12 to 24 months of observation period (month 24). All clinical parameters were determined at each patient visit or hospitalization and were recorded in our local patient registry.

**Table 1:** Demographic and baseline characteristics of the study group.

	*Total*	*Control*	*Tiotropium 18 μg*	*p value*
No. of patients, n (%)	160 (100.0)	80 (100.0)	80 (100.0)	
Male sex, n (%)	62 (38.8)	31 (38.8)	31 (38.8)	
Pancreatic insufficient, n (%)	151 (94.4)	75 (93.8)	76 (95.0)	>0.9999
Age, year, mean ± SD	31.3 ± 9.3	30.5 ± 8.9	32.0 ± 9.8	0.4261
BMI, kg/m^2^, mean ± SD	20.2 ± 3.5	19.7 ± 3.6	20.6 ± 3.3	0.2144
Mutation, n (%)				
dF508/dF508	65 (40.6)	27 (33.8)	38 (47.5)	0.1071
dF508 heterozygous	62 (38.8)	33 (41.3)	29 (36.3)	0.6266
other	33 (20.6)	20 (25.0)	13 (16.3)	0.2408
Percent-predicted FEV1, mean ± SD	54.1 ± 20.9	54.7 ± 20.7	53.5 ± 21.1	0.6986
Percent-predicted FEV1 group, n (%)				
FEV1_0M_ ≥70%	38 (23.8)	19 (23.8)	19 (23.8)	
FEV1_0M_ 50–69%	44 (27.5)	22 (27.5)	22 (27.5)	
FEV1_0M_ ≤49%	78 (48.8)	39 (48.8)	39 (48.8)	
Tiotropium medication, n (%)	80 (50.0)	0 (0.0)	80 (100.0)	
Baseline concomitant medication, n (%)				
Inhaled antibiotics	128 (80.0)	65 (81.3)	63 (78.8)	0.8436
Long-acting β_2_ agonists	89 (55.6)	40 (50.0)	49 (61.3)	0.2029
Inhaled glucocorticoids	10 (6.3)	5 (6.3)	5 (6.3)	>0.9999
Systemic glucocorticoids	21 (13.1)	9 (11.3)	12 (15.0)	0.6405
*Pseudomonas aeruginosa* positive, n (%)	128 (80.0)	65 (81.3)	63 (78.8)	0.8436

Values expressed as mean ± standard deviation (SD) and number of patients (n) and proportion (%). BMI: body mass index, FEV1: forced expiratory volume in 1 second, FEV1_0M_: baseline FEV1 equates to begin (month 0) of observation period and before tiotropium treatment started.

The index date, for patients treated with tiotropium, was defined as date of tiotropium prescription. The index date indicated the last patient visit before first tiotropium intake. Baseline characteristics, including FEV1_0M_ as a matching variable, were determined at the index date. The 80 selected controls were identified via matching variables (gender, age ± 5 years, FEV1_0M_ ± 5%). The index date, for tiotropium untreated patients, was defined as date of first occurrence of the index event (FEV1_0M_ ± 5%, gender, age ± 5 years) during both the patient′s enrollment period and the analysis period (2004 to 2014).

The main outcome in this study was the mean annual change of FEV1 from index date until the end of a 24 month period of observation.

### Study subjects

A total of 415 patients were recorded in our local patient registry from 2004 to 2014 and each of the eligibility criteria below was examined. Inclusion criteria were: ≥18 years of age, patients have given written informed consent, patients with confirmed CF diagnosis, pulmonary lung function measures for a 24 months observation period and measures of exacerbation for 24 months. Exclusion criteria were: patients after lung transplantation, patients that did not meet matching criteria and patients with <5 FEV1 measures during observation period. Finally, 160 patients, aged 18–61 years, were included in our study with 80 patients per group (tiotropium treated and tiotropium untreated). The matching criteria were: gender, age ± 5 years and FEV1_0M_ ± 5%. A number of 135 patients out of 415 patients with CF were treated with tiotropium and 280 patients out of 415 patients were never treated with tiotropium. Due to our inclusion, exclusion and matching criteria 55 patients of 135 tiotropium treated patients were excluded and 200 patients of non-tiotropium treated 280 patients were excluded. A matched-pair analysis comparing patients who underwent tiotropium treatment and those not treated with tiotropium was performed. The matching ratio was 1:1. All patients were grouped into three different disease severities on the basis of baseline FEV1/FEV1_0M_ at the beginning (month 0) of 24 months observation period and before tiotropium treated patients start their tiotropium add-on therapy. The subgroup determination was similar to those reported elsewhere [10]. Pre-bronchodilator baseline FEV1_0M_ ≥70% predicted for a mild, FEV1_0M_ 50–69% predicted for a moderate and a moderately severe and FEV1_0M_ ≤49% predicted for a severe and a very severe degree of CF lung disease severity [10]. Tiotropium was used as add-on to standard CF treatment. Patients received 18 μg of tiotropium once daily, delivered through the HandiHaler powder formulation inhalation device (Boehringer Ingelheim, Germany). All patients were able to inhale tiotropium and they were routinely educated in inhalation technique. Compliance was reviewed and documented at each visit. All patients proceeded to receive their standard-of-care therapy during the observed time period. The concomitant use of pulmonary medication, using short- and long-acting β-agonists as well as short acting anticholinergic drugs, was allowed. The number of study subjects treated with β-agonists and/or glucocorticoids and inhaled antibiotics did not differ between the groups (Table 1).

### Statistical analysis

In this retrospective cohort study, 415 patients with CF were screened. Due to eligibility criteria 160 patients with CF were included. The matching ratio was 1:1, in which pairs of tiotropium treated and tiotropium untreated subjects were formed. To estimate an adequate sample size we focused on the FEV1 change between groups. A mean change of -1.44 in the control group and a mean change of 0.78 in the tiotropium group for FEV1 were estimated [24, 28–30]. By assuming a common standard deviation of 4.8 for both groups, an effect size of 0.46 was calculated [24, 28–30]. A two-sided t-test and a sample size of 80 per group lead to a Power of 82% [31]. We applied an alpha of 0.05. Results were presented as means ± standard deviations (SD) or median and interquartile range (IQR) as appropriate, unless otherwise stated. The characteristics of tiotropium treated patients versus non-tiotropium treated control patients were compared, using non-parametric, non-paired Mann-Whitney U or t–test depending on scale and distribution. For comparison of different severity groups, FEV1_0M_: ≥70%, 50–69% and ≤49%, of control and tiotropium groups, the Kruskal-Wallis test was used. Furthermore, mean annual change of FEV1 was adjusted for confounding using multiple linear regression.

GraphPad Prism (GraphPad Software, Inc., La Jolla, CA, USA) was used to perform statistical analysis. A *p* value < 0.05 was considered statistically significant.

## Results

### Cohort characteristics

**Fig 1:**
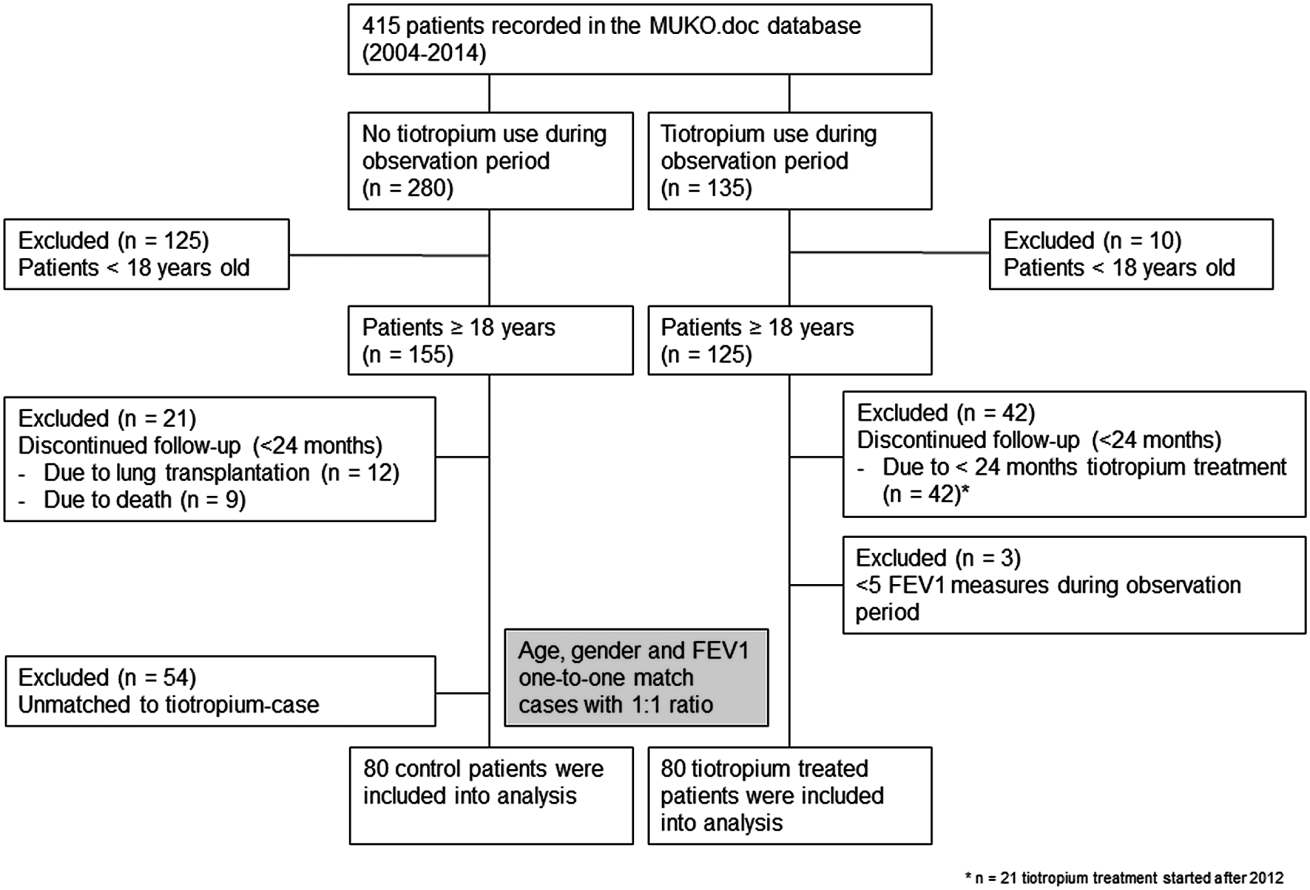
Flow chart of patient selection for tiotropium treated and non-tiotropium treated control patient comparison. All patients with confirmed CF diagnosis recorded in the MUKO.doc database have given written informed consent.

This retrospective cohort analysis included longitudinal data of 160 patients with CF, registered in MUKO.doc database from February 2004 until July 2014. Patients with less than 24 months tiotropium treatment were excluded (Fig 1). Two cases of a dry mouth symptom, typically associated with anticholinergics were reported in the tiotropium treated group but did not lead to discontinuation of treatment.

The demographic data, mean age and baseline pulmonary function (FEV1_0M_) were comparable across the study groups and are summarized in Table 1. Furthermore, the multiple linear regression showed only a significant influence of the tiotropium medication with an estimate beta of 1.9 (SE = 0.7) and p = 0.008. All confounding factors (Table 1) including baseline FEV1_0M_ remained without significant impact.

### Annual change of lung function

**Fig 2:**
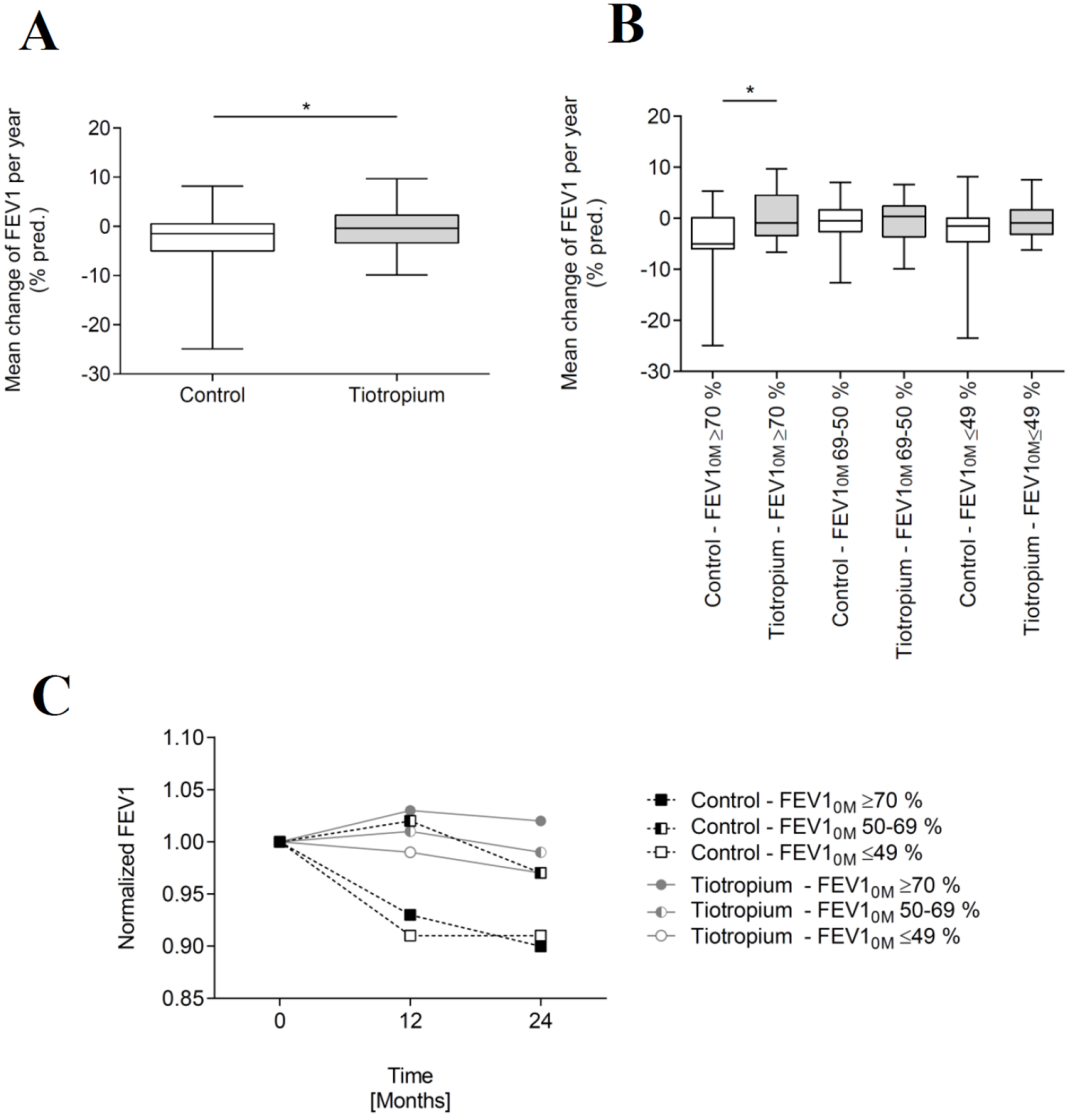
Annual change of FEV1. Mean change of FEV1 (percent predicted) per year for (A) control (n = 80) and tiotropium treated study group (n = 80; *p* = 0.0130) and (B) for different disease severities: FEV1_0M_ ≥70% (n = 19; *p* = 0.0132), FEV1_0M_ 50–69% (n = 22), FEV1_0M_ ≤49% (n = 39). (C) Normalized mean FEV1 values of tiotropium (n = 80) and non-tiotropium treated control patients (n = 80) and for different disease severities: FEV1_0M_ ≥70% (n = 19), FEV1_0M_ 50–69% (n = 22), FEV1_0M_ ≤49% (n = 39) on 0, 12 and 24 months observation period.

There was a significant difference between non-tiotropium treated control (n = 80; -2.3 ± 5.0% p.a.) and tiotropium treated study group (n = 80; -0.3 ± 4.0% p.a) in mean annual change of FEV1 percent predicted (*p* = 0.0130; Fig 2A and Table 2). Particularly, tiotropium treated patients with FEV1_0M_ ≥70% showed significantly positive development of the mean annual change of FEV1 compared to untreated control patients (control: n = 19; -4.0 ± 6.3% p.a.; tiotropium: n = 19; +0.5 ± 4.7% p.a.; *p* = 0.0132; Fig 2B and Table 2). The demographic data and baseline characteristics were comparable across the tiotropium treated and non-tiotropium treated control subgroups with baseline FEV1_0M_ ≥70% and are summarized in S1 Table.

**Table 2:** Outcomes by allocation.

	*Control*	*Tiotropium 18 μg*	*p Value*
Mean annual change of Percent-predicted FEV1, mean ± SD (n)	-2.3 ± 5.0% (80)	-0.3 ± 4.0% (80)	0.0130
FEV1_0M_ ≥70%	-4.0 ± 6.3% (19)	+0.5 ± 4.7% (19)	0.0132
FEV1_0M_ 50–69%	-0.8 ± 3.8% (22)	-0.5 ± 4.4% (22)	0.7142
FEV1_0M_ ≤49%	-2.4 ± 4.8% (39)	-0.6 ± 3.4% (39)	0.0898
Mean change of exacerbations per year,mean ± SD (n)	+0.2 ± 1.7 (80)	+0.1 ± 1.9 (80)	0.6482
FEV1_0M_ ≥70%	-0.3 ± 1.5 (19)	+0.3 ± 1.4 (19)	0.3818
FEV1_0M_ 50–69%	+0.80 ± 1.1 (22)	0.0 ± 2.1 (22)	0.1481
FEV1_0M_ ≤49%	+0.1 ± 2.1 (39)	+0.1 ± 2.0 (39)	0.9266

Values expressed as mean ± standard deviation (SD) and number of patients (n). FEV1: forced expiratory volume in 1 second, FEV1_0M_: baseline FEV1 equates to begin (month 0) of observation period and before tiotropium treatment started.

Tiotropium treated patients with FEV1_0M_ lower than 70% indicated smaller amounts but not statistically significant for annual lung function decline compared to untreated control patients.

More precisely, tiotropium treated patients with a moderate and moderately severe degree of severity (FEV1_0M_ 50–69%) were characterized by smaller absolute values for mean annual change of FEV1 compared to control patients (control: n = 22; -0.8 ± 3.8% p.a.; tiotropium: n = 22; -0.5 ± 4.4% p.a.; *p* = 0.7142; Fig 2B and Table 2). Baseline characteristics were comparable across tiotropium untreated and tiotropium treated patients with baseline FEV1_0M_ 50–69% and are summarized in S2 Table.

A similar observation for mean change of lung function per year (p.a.) was noted in patients with a severe and very severe degree of severity, i.e. ≤ 49% baseline FEV1_0M_ values (control: n = 39; -2.4 ± 4.8% p.a.; tiotropium: n = 39; -0.6 ± 3.4% p.a.; *p* = 0.0898; Fig 2 and Table 2; please see S3 Table for baseline characteristics).

The rate of lung function decline for different disease severities on 12 and 24 months of observation period are shown in Fig 2C. Patients with FEV1_0M_ ≥70% and ≤49% without tiotropium therapy had continuous lower FEV1 values compared to baseline and to tiotropium treated study group. In the subgroup of patients with a moderate and moderately severe degree of severity (FEV1_0M_ 50–69%), there were comparable lung functions for untreated and treated patients until 12 months of observation period. After >12 months of treatment, the tiotropium group showed slightly higher FEV1 values compared to untreated controls. Furthermore, tiotropium treatment resulted in continuous higher FEV1 values compared to baseline for patients with CF with FEV1_0M_ ≥70%.

### Pulmonary Exacerbations

There was no statistical difference between tiotropium and untreated study group for mean annual change of exacerbations (control: n = 80; +0.2 ± 1.7% p.a.; tiotropium: n = 80; +0.1 ± 1.9% p.a.; *p* = 0.6482; Table 2). Regarding different disease severities, FEV1_0M_ ≥70%, 50–69% and ≤49% are shown in Table 2. Tiotropium treatment was not associated with changes in mean annual change of exacerbation in the observed study cohort.

## Discussion

In the presented retrospective cohort study, for the first time, data on long-term tiotropium therapy (24 months, 18 μg) used in the pulmonary treatment of adult patients with CF are provided. The results suggest a reduced decline rate for mean annual change of FEV1, without influencing mean annual change of exacerbation in adult patients with CF.

As compared with findings of a previous phase II trial in patients with CF [24], the results shown here (mean change of FEV1 percent predicted: control: -2.3 ± 5.0% p.a.; n = 80; tiotropium: -0.3 ± 4.0% p.a.; n = 80; *p* = 0.0130; Fig 2A) support that once-daily inhaled tiotropium reduces rates of lung function decline statistically significant. With regard to existing data focused on bronchodilator utilization in COPD patients, it is known that tiotropium treatment (≥ 12 months) is effective in improving lung function [19–22]. The mean rate of FEV1 decline in our control cohort was similar to those reported elsewhere [32]. In contrast, results of a phase III trial with 12 weeks of tiotropium treatment in CF [25] could not confirm statistically significant improvements in lung function induced by tiotropium. The observed period of 12 weeks tiotropium treatment [24, 25] reflects a smaller time period and may not adequately monitor long-term modulatory effects of tiotropium treatment in CF. Tashkin and coworkers claimed that short-term bronchodilator response was not an adequate criterion to predict long-term outcome of FEV1 course [19]. According to this, tiotropium therapy duration seemed to be crucial for therapy success. In our study, a higher dosage of 18 μg tiotropium has been applied, instead of 2.5 and 5 μg tiotropium used in former studies [24, 25]. The improvement in lung function was larger with higher tiotropium dosages [24]. Similar to our results, studies among patients with COPD, dosages of 18 μg tiotropium resulted in reduced lung function decline [19–22]. Beside tiotropium dosage other factors, such as selection of certain patient groups (e.g. age) and study design could also account for differences.

We provide long-term tiotropium information on different FEV1 groups at baseline. Tiotropium treatment seemed to be most effective in patients with a mild degree of CF disease severity: FEV1_0M_ ≥70% (Fig 2B and 2C). Based on this finding, we hypothesized that, patients with lower FEV1_0M_ values had advanced airway destruction and fixed obstruction, resulting in partly restricted admission of inhaled tiotropium. Pulmonary exacerbations are promoted by inflammation and negatively influenced mucociliary clearance, which contribute to a worsening airway obstruction, a decline in lung function, and a profound negative impact on perceived quality of life [2]. As pulmonary exacerbations have very severe consequences in CF, both in terms of current morbidity as well as implications for long-term morbidity and mortality, the reduction of exacerbation risk is one goal of CF treatment [2, 33]. Here, tiotropium treatment was not associated with modifications in mean change of exacerbation. Related findings have been reported elsewhere [25]. Since 2004, 135 of 415 patients with CF received tiotropium treatment in our center. Eighty adult patients with CF have been treated with a long-term tiotropium therapy and were included in this study. Tiotropium is well tolerated in patients with CF consistent with the known safety profile in COPD [25, 34].

This study provides data on the efficacy of long-term tiotropium treatment administered for 24 months to adult patients with a wide range of baseline FEV1_0M_. In order to minimize bias and confounding, a matching procedure on potential confounders was performed. Limitations of this study include the relatively small number of patients as a consequence of CF being a rare disease, the susceptibility of the study design for confounding and bias, especially selection and observer bias. Moreover, it should be taken into account, that this study was neither randomized nor placebo controlled due to the fact that all parameters in this study have been assessed retrospectively. Another potential limitation relates to the complex standard CF medication regimes including multiple drugs and application intervals.

Further prospective and randomized long-term studies of tiotropium treatment in CF are necessary. This study may represent a pilot study to evaluate possible parameters and procedures for further evaluations. Patients with CF showed altered airway smooth muscle physiology resulting in airway smooth muscle contraction, airway narrowing and airway dysfunction [35]. We hypnotized that the pathophysiological reasons of clinical benefits with tiotropium are various: tiotropium is an anticholinergic agent, able to reduce basal airway smooth muscle tone, it has the potential to inhibit allergen-induced increases in airway smooth muscle thickening in animal models [36] and in addition to its effect as a bronchodilator [37] tiotropium might have an inhibitory effect on airway remodeling in various non-CF animal models [6, 38, 39]. Furthermore, it was suggested that a prolonged duration of treatment with tiotropium was required for beneficial effect in animal models [39]. Airway epithelium damage and remodeling are fundamental elements of lung pathology progression in CF [40]. Therefore, long-term tiotropium could be an additional effective treatment option to relieve bronchoconstriction and to prevent airway remodeling in the future [39].

Continuative research is needed to elucidate the positive effects of long-term anti-obstructive tiotropium therapy, safety and tolerability in patients with CF and to identify differences between different CF patient groups, e.g. disease severities. The optimization of CF standard therapy regarding anti-obstructive therapy will be the objective for further studies.

## Supporting Information

S1 Table Demographic and baseline characteristics of the study group with FEV1_0M_ ≥70%. (PDF)

S2 Table Demographic and baseline characteristics of the study group with FEV1_0M_ 50–69%. (PDF)

S3 Table Demographic and baseline characteristics of the study group with FEV1_0M_ ≤49%. (PDF)

S4 Table Outcomes by allocation. (PDF)

## Abbreviations

CF: Cystic Fibrosis
FEV1: forced expiratory volume in 1 s
COPD: chronic obstructive pulmonary disease
