# Supplementary material for: Impact of Long-Term Tiotropium Bromide Therapy on Annual Lung Function Decline in Adult Patients with Cystic Fibrosis
Source: PLoS One. 2016 Jun 28;11(6):e0158193. doi: 10.1371/journal.pone.0158193 (PMC4924629; doi:10.1371/journal.pone.0158193)
Supplement: S1 Table — (PDF) [file pone.0158193.s001.pdf]

S1 Table. Demographic and baseline characteristics of the study group with FEV1$_{0M}$ ≥70 %.

Subgroup FEV1$_{0M}$ ≥70 %	***Total***	***Control***	***Tiotropium 18 µg***	***p value***
No. of patients, n (%)	38 (100.0)	19 (100.0)	19 (100.0)	
Male sex, n (%)	12 (31.6)	6 (31.6)	6 (31.6)	
Pancreatic insufficient, n (%)	34 (89.5)	17 (89.5)	17 (89.5)	> 0.9999
Age, year, mean ± SD	29.4 ± 7.2	28.3 ± 6.7	30.4 ± 7.8	0.4652
BMI, kg/m^2, mean ± SD	21.4 ± 2.9	21.8 ± 3.1	20.9 ± 2.7	0.4653
Mutation, n (%)				
dF508/dF508	15 (39.5)	6 (31.6)	9 (47.4)	0.5077
dF508 heterozygous	18 (47.4)	10 (52.6)	8 (42.1)	0.7459
other	5 (13.2)	3 (15.8)	2 (10.5)	> 0.9999
Percent-predicted FEV1, mean ± SD	83.8 ± 8.1	84.1 ± 8.5	83.6 ± 7.9	0.8453
Percent-predicted FEV1 group, n (%)				
FEV1$_{0M}$ ≥70 %	38 (100.0)	19 (100.0)	19 (100.0)	
FEV1$_{0M}$ 50-69 %	0 (0.0)	0 (0.0)	0 (0.0)	
FEV1$_{0M}$ ≤49 %	0 (0.0)	0 (0.0)	0 (0.0)	
Tiotropium medication, n (%)	19 (50.0)	0 (0.0)	19 (100.0)	
Baseline concomitant medication, n (%)				
Inhaled antibiotics	30 (78.9)	16 (84.2)	14 (73.7)	0.6928
Long-acting β_2 agonists	24 (63.2)	15 (78.9)	9 (47.4)	0.0911
Inhaled glucocorticoids	6 (15.8)	3 (15.8)	3 (15.8)	> 0.9999
Systemic glucocorticoids	4 (10.5)	0 (0.0)	4 (21.1)	0.105
Pseudomonas aeruginosa positive, n (%)	30 (78.9)	16 (84.2)	14 (73.7)	0.6928

Values expressed as mean ± standard deviation (SD) and number of patients (n) and proportion (%).
BMI: body mass index, FEV1: forced expiratory volume in 1 second, FEV1$_{0M}$: baseline FEV1 equates to begin (month 0) of observation period and before tiotropium treatment started.
