# Supplementary material for: Impact of Long-Term Tiotropium Bromide Therapy on Annual Lung Function Decline in Adult Patients with Cystic Fibrosis
Source: PLoS One. 2016 Jun 28;11(6):e0158193. doi: 10.1371/journal.pone.0158193 (PMC4924629; doi:10.1371/journal.pone.0158193)
Supplement: S2 Table — (PDF) [file pone.0158193.s002.pdf]

S2 Table. Demographic and baseline characteristics of the study group with $FEV1_{0M}$ 50-69 %.

Subgroup $FEV1_{0M}$ 50-69%	*Total*	*Control*	*Tiotropium 18 µg*	*p value*
No. of patients, n (%)	44 (100.0)	22 (100.0)	22 (100.0)	
Male sex, n (%)	22 (50.0)	11 (50.0)	11 (50.0)	
Pancreatic insufficient, n (%)	42 (95.5)	21 (95.5)	21 (95.5)	> 0.9999
Age, year, mean ± SD	34.3 ± 10.7	33.4 ± 10.7	35.2 ± 10.9	0.5968
BMI, kg/m^2, mean ± SD	20.9 ± 4.5	20.0 ± 5.1	21.8 ± 3.7	0.5647
Mutation, n (%)				
dF508/dF508	17 (38.6)	7 (31.8)	10 (45.5)	0.5365
dF508 heterozygous	17 (38.6)	9 (40.9)	8 (36.4)	> 0.9999
other	10 (22.7)	6 (27.3)	4 (18.2)	0.7205
Percent-predicted FEV1, mean ± SD	59.5 ± 6.6	59.9 ± 7.4	59.1 ± 5.7	0.8386
Percent-predicted FEV1 group, n (%)				
$FEV1_{0M}$ ≥70 %	0 (0.0)	0 (0.0)	0 (0.0)	
$FEV1_{0M}$ 50-69 %	44 (100.0)	22 (100.0)	22 (100.0)	
$FEV1_{0M}$ ≤49 %	0 (0.0)	0 (0.0)	0 (0.0)	
Tiotropium medication, n (%)	22 (50.0)	0 (0.0)	22 (100.0)	
Baseline concomitant medication, n (%)				
Inhaled antibiotics	33 (75.0)	18 (81.8)	15 (68.2)	> 0.9999
Long-acting β_2 agonists	24 (54.5)	11 (20.0)	13 (59.1)	0.7626
Inhaled glucocorticoids	2 (4.5)	1 (4.5)	1 (4.5)	> 0.9999
Systemic glucocorticoids	3 (6.8)	2 (9.1)	1 (4.5)	> 0.9999
Pseudomonas aeruginosa positive, n (%)	33 (75.0)	18 (81.8)	15 (68.2)	> 0.9999

Values expressed as mean ± standard deviation (SD) and number of patients (n) and proportion (%).

BMI: body mass index, FEV1: forced expiratory volume in 1 second, $FEV1_{0M}$: baseline FEV1 equates to begin (month 0) of observation period and before tiotropium treatment started.
