# Supplementary material for: Impact of Long-Term Tiotropium Bromide Therapy on Annual Lung Function Decline in Adult Patients with Cystic Fibrosis
Source: PLoS One. 2016 Jun 28;11(6):e0158193. doi: 10.1371/journal.pone.0158193 (PMC4924629; doi:10.1371/journal.pone.0158193)
Supplement: S3 Table — (PDF) [file pone.0158193.s003.pdf]

S3 Table. Demographic and baseline characteristics of the study group with $FEV1_{0M}$ ≤49 %.

Subgroup $FEV1_{0M}$ ≤49 %	***Total***	***Control***	***Tiotropium 18 µg***	***p value***
No. of patients, n (%)	78 (100.0)	39 (100.0)	39 (100.0)	
Male sex, n (%)	28 (35.9)	14 (35.9)	14 (35.9)	
Pancreatic insufficient, n (%)	75 (96.2)	37 (94.9)	38 (97.4)	> 0.9999
Age, year, mean ± SD	30.5 ± 9.2	30.0 ± 8.6	31.0 ± 9.8	0.8639
BMI, kg/m^2, mean ± SD	19.1 ± 2.8	18.6 ± 2.2	19.7 ± 3.2	0.0417
Mutation, n (%)				
dF508/dF508	33 (42.3)	14 (35.9)	19 (48.7)	0.3594
dF508 heterozygous	27 (34.6)	14 (35.9)	13 (33.3)	> 0.9999
other	18 (23.1)	11 (28.2)	7 (17.9)	0.4207
Percent-predicted FEV1, mean ± SD	36.5 ± 8.7	37.4 ± 8.5	35.6 ± 9.0	0.335
Percent-predicted FEV1 group, n (%)				
$FEV1_{0M}$ ≥70 %	0 (0.0)	0 (0.0)	0 (0.0)	
$FEV1_{0M}$ 50-69 %	0 (0.0)	0 (0.0)	0 (0.0)	
$FEV1_{0M}$ ≤49 %	78 (100.0)	39 (100.0)	39 (100.0)	
Tiotropium medication, n (%)	39 (50.0)	0 (0.0)	39 (100.0)	
Baseline concomitant medication, n (%)				
Inhaled antibiotics	66 (84.6)	32 (82.1)	34 (87.2)	> 0.9999
Long-acting β_2 agonists	41 (52.6)	14 (35.9)	27 (69.2)	0.0061
Inhaled glucocorticoids	2 (2.6)	1 (2.6)	1 (2.6)	> 0.9999
Systemic glucocorticoids	14 (17.9)	7 (17.9)	7 (17.9)	> 0.9999
Pseudomonas aeruginosa positive, n (%)	66 (84.6)	32 (82.1)	34 (87.2)	> 0.9999

Values expressed as mean ± standard deviation (SD) and number of patients (n) and proportion (%).

BMI: body mass index, FEV1: forced expiratory volume in 1 second, $FEV1_{0M}$: baseline FEV1 equates to begin (month 0) of observation period and before tiotropium treatment started.
