# Supplementary material for: Impact of Long-Term Tiotropium Bromide Therapy on Annual Lung Function Decline in Adult Patients with Cystic Fibrosis
Source: PLoS One. 2016 Jun 28;11(6):e0158193. doi: 10.1371/journal.pone.0158193 (PMC4924629; doi:10.1371/journal.pone.0158193)
Supplement: S4 Table — (PDF) [file pone.0158193.s004.pdf]

S4 Table. Outcomes by allocation.

Mean number of exacerbation	***Control***	***Tiotropium 18 µg***	***p Value***
0-12 months of observation period, mean ± SD (n)			
$FEV1_{0M}$ ≥70%	1.4 ± 1.3 (19)	1.7 ± 1.5 (19)	0.5142
$FEV1_{0M}$ 50-69 %	1.0 ± 0.8 (22)	2.0 ± 1.5 (22)	0.0086
$FEV1_{0M}$ ≤49%	2.2 ± 1.6 (39)	2.6 ± 1.9 (39)	0.3178
12-24 months of observation period, mean ± SD (n)			
$FEV1_{0M}$ ≥70%	1.2 ± 1.2 (19)	1.9 ± 1.5 (19)	0.1209
$FEV1_{0M}$ 50-69 %	1.8 ± 1.2 (22)	1.9 ± 1.4 (22)	0.8004
$FEV1_{0M}$ ≤49%	2.4 ± 2.0 (39)	2.6 ± 1.8 (39)	0.6438

Values expressed as mean ± standard deviation (SD) and number of patients (n).
FEV1: forced expiratory volume in 1 second, $FEV1_{0M}$: baseline FEV1 equates to begin (month 0) of observation period and before tiotropium treatment started.
